# Magnetron Sputtering as a Fabrication Method for a Biodegradable Fe32Mn Alloy

**DOI:** 10.3390/ma10101196

**Published:** 2017-10-18

**Authors:** Till Jurgeleit, Eckhard Quandt, Christiane Zamponi

**Affiliations:** Chair for Inorganic Functional Materials, Institute for Materials Science, Faculty of Engineering, University of Kiel, Kaiserstrasse 2, 24143 Kiel, Germany; tiju@tf.uni-kiel.de (T.J.); eq@tf.uni-kiel.de (E.Q.)

**Keywords:** magnetron sputtering, biodegradable metals, FeMn alloys, material characterization

## Abstract

Biodegradable metals are a topic of great interest and Fe-based materials are prominent examples. The research task is to find a suitable compromise between mechanical, corrosion, and magnetic properties. For this purpose, investigations regarding alternative fabrication processes are important. In the present study, magnetron sputtering technology in combination with UV-lithography was used in order to fabricate freestanding, microstructured Fe32Mn films. To adjust the microstructure and crystalline phase composition with respect to the requirements, the foils were post-deposition annealed under a reducing atmosphere. The microstructure and crystalline phase composition were investigated by scanning electron microscopy, energy dispersive X-ray spectroscopy, and X-ray diffraction. Furthermore, for mechanical characterization, uniaxial tensile tests were performed. The in vitro corrosion rates were determined by electrochemical polarization measurements in pseudo-physiological solution. Additionally, the magnetic properties were measured via vibrating sample magnetometry. The foils showed a fine-grained structure and a tensile strength of 712 MPa, which is approximately a factor of two higher compared to the sputtered pure Fe reference material. The yield strength was observed to be even higher than values reported in literature for alloys with similar composition. Against expectations, the corrosion rates were found to be lower in comparison to pure Fe. Since the annealed foils exist in the austenitic, and antiferromagnetic γ-phase, an additional advantage of the FeMn foils is the low magnetic saturation polarization of 0.003 T, compared to Fe with 1.978 T. This value is even lower compared to the SS 316L steel acting as a gold standard for implants, and thus enhances the MRI compatibility of the material. The study demonstrates that magnetron sputtering in combination with UV-lithography is a new concept for the fabrication of already in situ geometrically structured FeMn-based foils with promising mechanical and magnetic properties.

## 1. Introduction

In the recent years, biodegradable metals have been the subject of intense research. Temporary medical implants such as wires, meshes, screws nails, and stents would be beneficial in order to reduce the risk of late complications such as stent restenosis and chronic inflammation reactions [1]. A biodegradable vascular implant has to keep its mechanical integrity to serve its purpose at least for the entire healing period of 3–12 months [2,3,4]. Afterwards, the degradation should occur as fast as possible. Several in vivo studies have shown that Fe is a suitable candidate for biodegradable cardiovascular implants. However, the degradation rate of pure Fe was found to be too slow [2,4]. Thus, the degradation rate either has to be accelerated or the mechanical strength has to be increased. A higher strength reduces the required thickness for implant structures to bear the load acting on the implant. As a consequence, even the surface-to-volume ratio is changed. Therefore, a lower corrosion rate can be compensated by a larger relative surface and thus reduce the time an implant is retained in the body.

For the use as medical implant, the magnetic properties are also of great importance. With respect to MRI investigations, a candidate material should exhibit a low magnetic polarization in order to reduce health risks due to magnetic-induced heating, forces, and torques on the implant. Furthermore, it prevents or reduces image artifacts due to susceptibility effects [5].

In the literature, numerous strategies were presented in order to obtain the desired material properties. Several studies investigated the microstructural influences on the mechanical properties and the degradation behavior of pure Fe. Therefore, different fabrication or metal processing techniques were investigated, such as electroforming (E-Fe) [6,7], magnetron sputtering (S-Fe) [8], rolling techniques [9,10], and equal channel angular pressing (ECAP-Fe) [11,12,13]. Also, the influence of implementing precipitates, namely Pd [14,15,16,17], Au [18,19,20,21], Pt [17], Fe_2_O_3_ particles [22], and carbon nanotubes [23] into the Fe matrix was studied. To enhance the material properties with respect to the requirements, the addition of different alloying elements such as Mn, Co, Al, W, Sn, B, C, and S was investigated [24]. Since Mn was found to show a sufficient biocompatibility and significantly enhance the mechanical properties, many studies focused on different compositions of FeMn alloys [15,25,26,27,28,29,30,31,32,33,34,35,36,37]. Even the magnetic properties of FeMn alloys were found to be highly beneficial for the intended use. If the Mn content in the alloy exceeds about 25 at %, the γ-phase becomes predominant, resulting in antiferromagnetic behavior [38,39]. FeMn alloys with an Mn content of 30–35 at % were found to show promising properties in terms of mechanical, magnetic, and corrosion behavior. In order to optimize the material properties, even the research on alternative fabrication methods is reasonable. Recent studies by Zamponi, de Miranda, and Siekmeyer et al. [40,41,42,43] showed that magnetron sputtering can be used to fabricate in situ structured and freestanding NiTi foils. Schlüter and Haffner et al. [44,45,46,47] demonstrated that this method can be used as well for freestanding, biodegradable Mg-based devices. It was shown that the technique allows the fabrication of scaffold devices with a film thickness up to 250 µm and minimal feature sizes of 5 µm [47].

In a previous work by the authors [8], potential biodegradable pure Fe foils were produced by the same technique. It was found that the foils show high strength due to the fine-grained, columnar crystalline structure. Since the magnetron sputtering technology is not limited to the deposition of pure metals, it can be used as well for the deposition of a wide variety of alloys. In the present study, magnetron sputtering was employed for the deposition of freestanding Fe32Mn (FeMn) foils geometrically structured by means of UV-lithography. The unique microstructure obtained by this technique enables the further enhancement of the mechanical properties. The microstructural properties and composition were investigated by scanning electron microscopy (SEM), X-ray diffraction (XRD), and energy dispersive X-ray spectroscopy (EDX). For the determination of the mechanical values, uniaxial tensile tests were performed. Furthermore, the in vitro degradation rates were measured by electrochemical polarization. Additionally, a vibrating sample magnetometer (VSM) was used in order to determine the magnetic properties of the foils. 

## 2. Materials and Methods

### 2.1. Sample Preparation

The preparation of the samples was done in a similar way as presented in the authors’ previous work [8,20]. An important difference, however, is the choice of the substrate material. In this work, monocrystalline, 4-inch Z-cut quartz wafers (Microchemicals) were used in order to minimize internal stresses arising due to the mismatch of the thermal expansion coefficients—α (α_α-Fe_ = 11.8 × 10^−6^ K^−1^ [48]; α_α-Mn_ = 21.7 × 10^−6^ K^−1^ [48]; α_γ-FeMn30_ ≈ 18 × 10^−6^ K^−1^ [49]; α_Si_ = 2.6 × 10^−6^ K^−1^ [48] α_α-SiO2_ ⊥Z = 13.7 × 10^−6^ K^−1^ [50])—and the elevated substrate temperatures *T*_S_ = 250 °C.

For the deposition of the in situ structured FeMn foils, the substrates first had to be pre-structured. Therefore, a Cu sacrificial layer was deposited by magnetron sputtering. All sputter depositions were done in a Von Ardenne CS730S cluster magnetron sputtering machine (VON ARDENNE, Dresden, Germany).

After the Cu deposition, the wafers received their final geometric structuring by UV-lithography followed by galvanic copper deposition [42]. For the final deposition, a 4-inch FeMn target (Ingpuls) with a nominal composition of 65 at % Fe, 35 at % and a purity of 99.9%, was used. The deposition parameters were 500 W, 25 sccm Ar flow, and a gas pressure of 2.3 × 10^−3^ mbar. The given parameters were chosen due to the experience of previous work in order to minimize the residual film stress, and this resulted in a sputter rate of approximately 2 nm·s^−1^. The final geometrical shape of the deposited films is predetermined by the structured substrate. To release these in situ structured films from the substrates, selective wet etching in an alkaline solution containing H_2_O, NH_3_, and H_2_O_2_ was done. The sample preparation was finished by annealing at 400 °C, 600 °C (only XRD samples), 800 °C or 950 °C for two hours respectively, in order to adjust the microstructure and the phase composition towards the needs. In order to achieve the desired ductility, the recrystallization temperature has to be reached (>600 °C). Furthermore, non-ferromagnetic behavior is required. Based on the below discussed findings by XRD in this study, only annealing temperatures that showed a significant reduction of α’-phase were considered for the further grain size determination, tensile tests, corrosion tests, and VSM measurements. Mn especially exhibits a very strong affinity to oxygen, thus, in order to prevent oxidation, very low O_2_ partial pressures in the range of 10^−24^ mbar [51] are required. Hence, the furnace recipient used for the annealing was first evacuated to a pressure in the 10^−5^ mbar range before annealing. Afterwards, the recipient was purged at 1 bar overpressure with a reducing gas mixture containing 90% and 10% H_2_ (VarigonH10^®^, Linde, Pullach, Germany). Additionally, an O_2_/H_2_O absorber (Oxisorb^®^, spectron, Frankfurt, Germany) immediately preceding the gas inlet was used to minimize gas contamination. After annealing, the samples were rapidly cooled down (0.7 °C/s) to room temperature under gas flow for one hour.

For the tensile tests, a “dog-bone” shaped sample design with a strut width of 0.5 mm, 7 mm strut length, 5.5 mm parallel length and a homogeneous thickness of 20 µm, was used. Square shaped samples with an edge length of 15 mm and 10 µm thickness were used for the corrosion and XRD measurements. Therefore, substrates were diced into pieces, followed by the deposition of the sacrificial layer. In order to prevent measurement artifacts due to shape anisotropy in the VSM, circular pieces with a radius of 2 mm were punched out of the 15 mm × 15 mm foils.

### 2.2. Microstructure

#### 2.2.1. X-ray Diffraction (XRD)

All XRD diffractograms were measured using a XRD-3000 PTS X-ray diffractometer (Seifert, Ahrensburg, Germany), employing monochromatic Cu-K𝛼 radiation. The θ-2 θ-absolute scans were performed in the range of 35° to 90°, with 0.05° step width and 3 s dwell time per step. Square-shaped samples with an edge length of 15 mm were employed. 

#### 2.2.2. Electron Microscopy (SEM/EDX)

Surface images of the samples were optically evaluated in order to determine the grain sizes of the different annealing states. To reveal the grains, focused ion beam (FIB) etching was used to prepare the sample surface. Also, the imaging was done using an FIB to get an additional orientation contrast of the grains due to ion channeling effects. Furthermore, images of the fracture area of the tensile test samples were made after the measurement to estimate the fracture behavior. EDX analyses were used to determine the composition of the sputtered foils. To determine the compositional homogeneity, an elemental mapping was done over an entire 4-inch wafer, coated with a 10 µm thick FeMn film. The measurements were done on a Helios NanoLab 600 (FEI, Frankfurt, Germany) and an EDX detector (Oxford instruments, Abingdon, UK).

### 2.3. Corrosion 

The electrochemical corrosion measurements were performed as previously reported [8], according to the ASTM G59–97 [52]. An electrolyte Hanks buffered salt solution (HBSS) (H1387, Sigma Aldrich, Taufkirchen, Germany) was modified with sodium bicarbonate 0.35 g/L. The pH of the solution was adjusted by CO_2_ inlet and held constant at 7.4 ± 0.05. The I(U) curves were measured using a three-electrode cell and a VersaStat 3 (Princton Applied Research). A Pt mesh was used as the counter electrode, an Ag/AgCl as the reference electrode, and the corrosion samples as the working electrode. The corrosion current density was determined by tafel extrapolation [53,54] in order to calculate the corrosion rates (*CR*) using Equation (1):

(1) $$CR=\frac{j_{c}M}{n\rho F}$$

where *j*_c_ = corrosion current density (Am^−2^), *ρ* = density 7690 kg/m^3^, *M* = molar mass 55 g/mol, *n* = 2 (number of elementary charges per reaction step), and *F* = Faraday constant. Based on investigations regarding the degradation mechanisms of Fe [55] and FeMn based alloys, [56] the anodic dissolution reaction follows Equations (2) and (3).

(2) $$Fe\to Fe^{2+}+2e^{-}$$

(3) $$Mn\to Mn^{2+}+2e^{-}$$

Since the surface roughness depends predominantly on the substrate roughness, no additional surface treatment was done, and the foils exhibit a mirror finished surface (𝑅_𝑎_ = 14 nm ± 3 nm).

In order to determine the mean value and deviation, four samples of each type were measured. 

### 2.4. Mechanical Properties

Uniaxial tensile tests were performed using a BETA 5 − 5/6 × 10 (Messphysik, Fürstenfeld, Austria) and the above described “dog-bone” shaped tensile test samples. The testing parameters were set to a strain rate of 0.4%/min and 60% force loss relative to the maximum applied force as fracture criterion. Samples annealed at 800 °C and 950 °C for two hours were measured. To express the results in stress, the sample dimensions’ strut width was measured by profilometer measurements while the thickness was determined with a dial gauge. Four samples were measured in order to determine the mean values and deviation of the yield strength (YS), ultimate tensile strength (UTS), and fracture strain (A).

### 2.5. Magnetic Properties

A vibrating sample magnetometer (VSM) of the type Lake Shore 7400 series was used to record magnetic polarization curves in order to determine saturation polarization *J*_S_, remanence *J*_R_, and coercive field *H*_C_. The measurements were performed in-plane in a range of ±0.5 T with a ramping rate of 3 mT s^−1^.

## 3. Results

### 3.1. Microstructure

**Figure 1:**
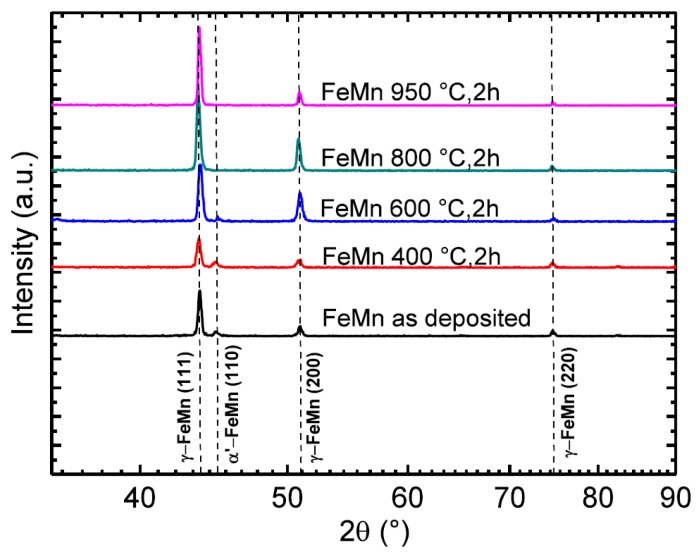
XRD measurements of freestanding FeMn foils as-deposited and after heat treatments at different temperatures for two hours.

In Figure 1, XRD diffractograms of freestanding FeMn foils after different heat treatments are shown. The (111) γ-FeMn reflection has the highest intensity. In all samples, even the (200) and (220) reflection corrosponding to the γ-FeMn phase can be found. The (110) reflex of the ferritic α’-phase is only present for samples annealed below 800 °C. After annealing at 600 °C, the intensity of the reflex decreases and finally disappears.

**Figure 2:**
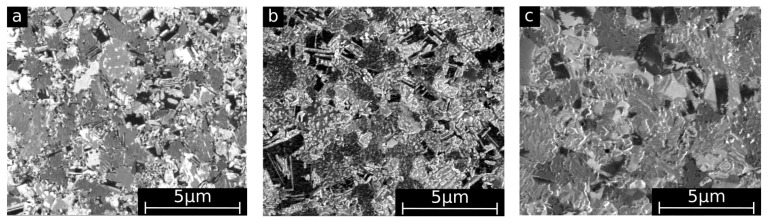
SEM/Focused ion beam (FIB) surface pictures of: (**a**) as-deposited; (**b**) annealed 800 °C for two hours; (**c**) annealed 950 °C for two hours.

**Figure 3:**
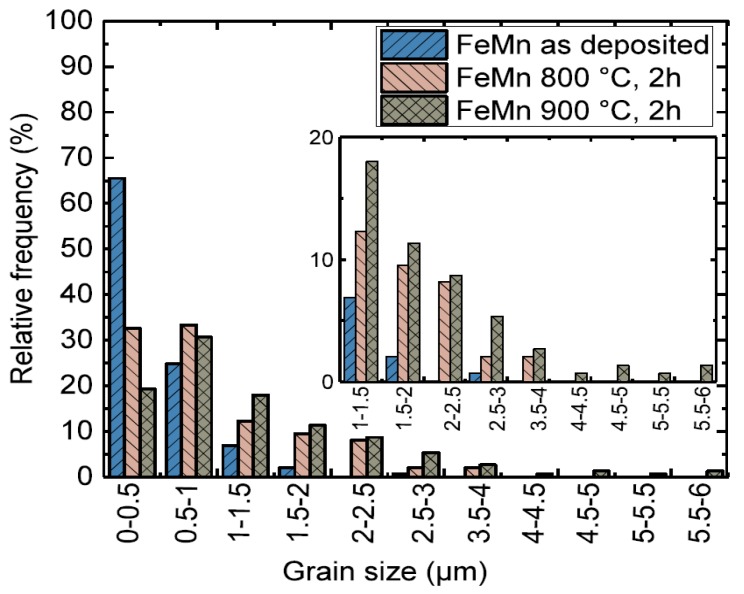
Grain size distribution of as-deposited and annealed FeMn samples.

**Figure 4:**
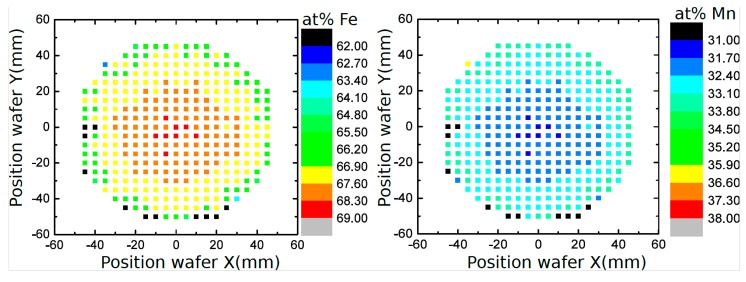
Composition distribution of the Fe and Mn over an entire 4-inch quartz wafer with a FeMn thickness of approximately 10 µm, measured by energy dispersive X-ray spectroscopy (EDX). The material is in the as-deposited state.

Figure 2 displays the SEM surface images used for the optical evaluation of the grain sizes. The images show a shift to larger grain sizes with increasing annealing temperature. Furthermore, the grains become more isometric after annealing, whereas in the as-deposited state numerous small needle shaped grains are also observed. In Figure 3, grain size distributions of as-deposited and annealed FeMn samples are shown. In Table 1, the mean grain size $\bar{d}$, maximum grain size *d*_max_, and minimum grain size *d*_min_ of as-deposited and annealed FeMn samples are summarized. The as-deposited films show a very fine-grained structure. The majority of the grains exhibit diameters below 500 nm. Whereas just a few grains show diameters >2 µm, several nano-scaled grains *d* <50 nm are observed. Annealing leads to a shift to larger grain diameters increasing with the temperature. However, even after annealing, a certain amount of grains *d* < 500 nm is present. Figure 4 displays the elemental distribution of the Fe and the Mn content over an entire 4-inch wafer, measured by EDX. Whereas in the center of the wafer, the composition is homogeneous in an area with a radius of approximately 25 mm (32 at % Mn and 68 at % Fe), in the outer regime, a shift ≈ 1 at % towards higher Mn contents is observed. The small tensile testing, XRD, corrosion, and VSM samples are taken from the center area with homogeneous composition. Furthermore, they are much smaller than the homogeneous region. Therefore, it is assumed that there is no elemental distribution within the single samples.

### 3.2. Corrosion

**Figure 5:**
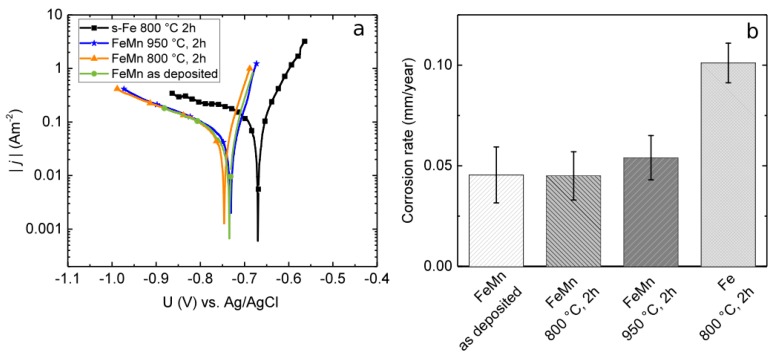
(**a**) Tafel plots of as-deposited, annealed FeMn, and annealed sputtered pure Fe; (**b**) Corrosion rates calculated from electrochemical measurements in Hanks buffered salt solution (HBSS) at 37 °C. For as-deposited and annealed FeMn samples in comparison to previously presented results for annealed pure Fe [8].

Exemplary measured tafel plots of as-deposited, annealed FeMn, and annealed sputtered pure Fe are shown in Figure 5a. No significant influence on *j*_c_ and the corrosion potential *U*_c_ between the FeMn samples are observed. In comparison to the pure Fe reference, the *j*_c_ values of all FeMn samples are approximately 0.05 Am^−2^ lower. The *U*_c_ values of the FeMn samples are approximately 80 mV negatively shifted in comparison to the pure Fe.

The mean values and deviations of the corrosion rates, calculated from the corrosion current densities using Equation (1), are shown in Figure 5b. In addition, a comparison value of the previously presented [8] sputtered pure Fe is shown. The FeMn samples exhibit a corrosion rate by the factor of two lower in comparison to the pure Fe. No significant influence of the heat treatments on the corrosion rates was found.

### 3.3. Mechanical Properties

**Figure 6:**
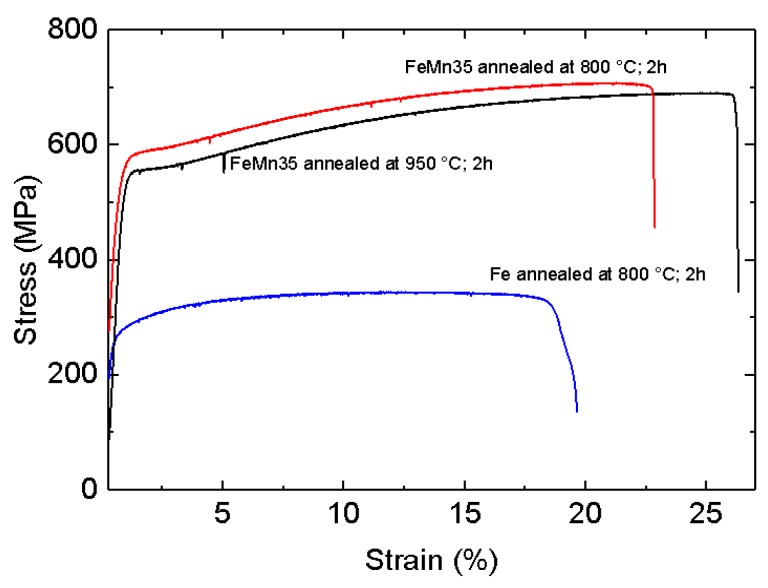
Exemplary stress–strain curves of annealed FeMn samples in comparison to previously presented results for annealed pure Fe [8].

**Figure 7:**
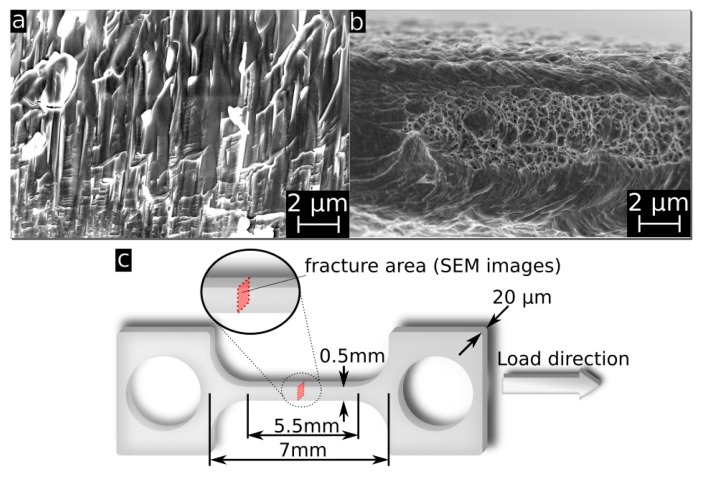
SEM image of the fracture surface of (**a**) as-deposited FeMn sample; (**b**) 800 °C annealed tensile testing sample; and (**c**) schematic drawing of a “dog-bone” shaped tensile testing sample with the relevant sample dimensions and an indication of the perspective of the SEM images.

**Figure 8:**
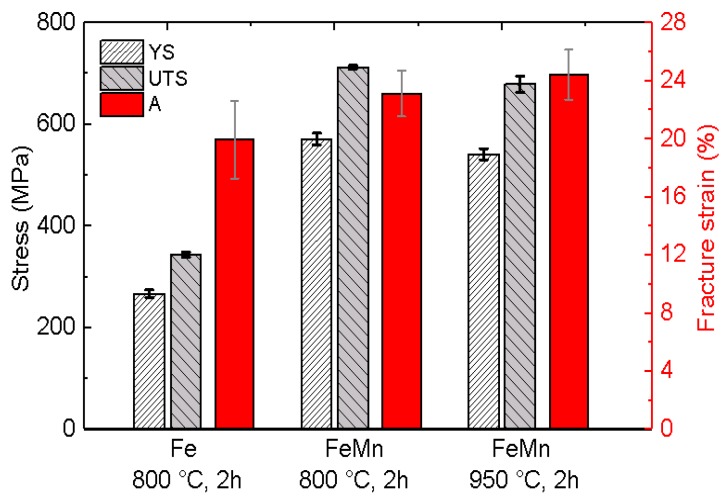
Mean values of the mechanical properties yield strength (YS), ultimate tensile strength (A) and fracture strain (A), of as-deposited and annealed FeMn samples in comparison to previously presented results for annealed pure Fe [8].

Exemplary stress-strain curves of annealed FeMn samples in comparison to previously presented results for annealed pure Fe are shown in Figure 6. Due to the markedly brittle behavior of the as-deposited samples, most of them were damaged during clamping. Therefore, it was not possible to obtain reliable results for the as-deposited samples. Figure 7a,b show the fracture surface of an as-deposited and an 800 °C annealed FeMn sample after the tensile test. The images show a clear difference in the fracture mode. The fracture area of the as-deposited sample shows columnar splintery areas which are visible in the vertical direction. This is also the growing direction during sputtering. After annealing, a dimple patterned fracture surface with necking is observed. The mean values and deviations of the tensile tests are summarized in Figure 8. Reference values for annealed sputtered pure Fe are given as well. Both the 800 °C and 950 °C annealed FeMn samples show an appreciably higher yield strength (YS) and ultimate tensile strength (UTS) compared to pure Fe. However, the samples annealed at 950 °C show a marginally lower strength. The fracture strain of the FeMn samples is approximately 4% higher than the values for pure Fe and increasing at higher annealing temperatures.

### 3.4. Magnetic Properties

**Figure 9:**
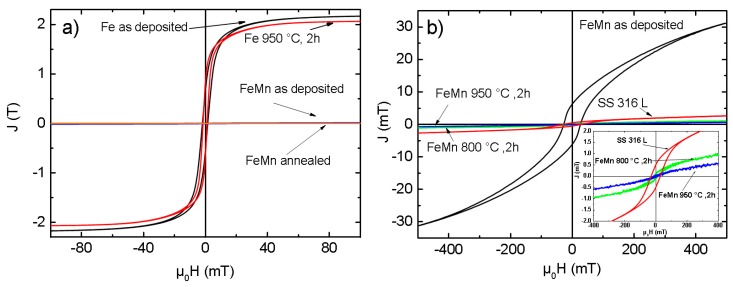
(**a**) Magnetic polarization curves of sputtered Fe and FeMn samples as-deposited and annealed at 950 °C for two hours; (**b**) Magnetic polarization curves of an SS 316L steel reference and sputtered FeMn samples as-deposited and annealed at different temperatures. The inset shows a zoom of the region of interest.

In Figure 9a, magnetic polarization curves of pure Fe in comparison with FeMn are shown for the as-deposited and annealed state. Figure 9b displays the polarization curves of sputtered FeMn as-deposited and in different annealing states compared to a SS 316L reference sample. The *J*_S_, *J*_R_, and *H*_c_ values for all samples are summarized in Table 2. Pure Fe, in comparison to FeMn and SS 316L, shows a several orders of magnitude higher magnetic saturation polarization. Whereas the *J*_S_ of the as-deposited FeMn is significantly higher compared to SS 316L, an explicit decrease of the polarization is observed for the annealed samples, even noticeable below the value for SS 316L.

## 4. Discussion

Magnetron sputtering in combination with UV-lithography is a feasible method to produce freestanding, structured FeMn foils. The grain size measurements (Figure 2 and Figure 3, Table 1) and XRD measurements (Figure 1) indicate a fine-grained microstructure composed of a fcc γ-FeMn phase and a Fe-rich bcc α’-phase. It was found that the phase composition of sputter-deposited films can distinctly differ from the thermodynamic equilibrium (TDE) and strongly depends on the composition [57]. During the deposition process, atoms condense on the substrate and undergo a random walk by diffusion. What type of nuclei and microstructure are formed mainly depends on the ratio of Mn to Fe atoms and substrate temperature *T*_S_. Studies on the dependency of *T*_S_ on the microstructure of sputtered films [58,59] showed that increasing *T*_S_ results in a microstructure more similar to those grown close to TDE. Thus, the elevated substrate temperature (250 °C) and the high Mn content in the present study result in a favored formation of the fcc γ-FeMn phase. However, both the XRD measurements and the slightly ferromagnetic nature determined by VSM indicate a certain amount of α’-FeMn phase. Since all initially observed reflexes are still present after annealing at 400 °C, there seems to be no significant changes in the crystalline phase composition. However, annealing at 600 °C leads to a reduction of the (110) α’ reflection and an increase of the γ reflections. After annealing at 800 °C and higher temperatures, only γ reflexes are observable. The equilibrium phase diagram of FeMn [60] exhibits an extensive γ region with a solubility for Mn up to 64 at % at 800 °C and 67 at % at 950 °C, respectively. During annealing, the high Mn concentration and increased diffusion processes lead to the stabilization of the γ-phase. The fast cooling after annealing inhibits the retransformation of new α’ grains during cooling. Since the amount of α’-phase is proportional to *J*_S_, it can be concluded that the observed stabilization of the γ-phase after annealing at temperatures above 600 °C is supported by the VSM data (Table 2, Figure 9). As suggested by the XRD investigation, the mean value of *J_S_* of the measured pure Fe (2.089 T) corresponds to 100 vol % ferromagnetic α’-phase. Furthermore, the low magnetic polarization arising from antiferromagnetic material can be neglected. In this case, the following α’-phase contents are estimated: FeMn as-deposited ≈1.49 vol %, FeMn 800 °C, 2 h ≈ 0.05 vol %, FeMn 950 °C, 2 h ≈ 0.03 vol %. 

The observed fine-grained structure is characteristic for sputtered materials (Table 1, Figure 2 and Figure 3). It is known that the film growth at a low ratio of substrate temperature to melting temperature *T*_S_/*T*_m_ < 0.3 is determined by the surface diffusion. The low surface diffusion hinders adatoms to overcome the roughness or forming nuclei. As a consequence, especially in the grain boundary regions, shadowing effects lead to a fine-grained and very defect rich structure, distinctive for sputtered films at low substrate temperatures [59,61]. Due to the high amount of defects, the movement of dislocations and plastic deformation is strongly limited.

Recrystallization is expected at annealing temperatures above 600 °C, according to the rule of thumb *T*_recrystallization_ ≈ 0.4 *T*_m_ with *T*_m_ ≈ 1300 °C [60]. This is in good agreement with the findings made in this study. Up to 600 °C, there is no evidence for a change in the crystalline phase composition (Figure 1) and thus a recrystallization. At 800 °C, there is a clear change of the crystalline phase composition indicated by XRD. Furthermore, the grain size distribution (Figure 3) shows a clear shift towards higher diameters with increasing annealing temperature. However, after annealing at 950 °C, there is still a very fine-grained structure present with a mean grain size in the lower µm range. Although a significant number of very small grains far below one µm are observed.

The observed brittleness of the as-deposited foils fits to the observed intercrystalline fracture mode (Figure 7a) and supports the assumption of a very defect rich growth, especially in the grain boundaries. A second reason for the initially low ductility is the amount of α’-phase evidenced by XRD. If α’ grains are dispersed in the γ-matrix, they act as further obstacles for dislocation movement and plastic deformation, respectively. 

Due to the drastically reduced defect density and predominance of the γ-phase, the annealed material exhibits high strength and concurrently high ductility. Whereas the high UTS has its origin in the intense solid solution hardening, there is also a strain hardening of ≈100 MPa observed in the strain range of 2–15% (Figure 6). The combination of high strength and ductility is characteristic for FeMn alloys with high Mn content. It is either attributed to a strain-induced transformation of the austenitic γ-phase into the martensitic ε-phase (TRIP effect) [62,63], or an intensive formation of strain-induced twins (TWIP effect) [63,64]. Both effects result in high tensile strength and ductility. Due to the composition used in this study, the TWIP effect seems to be more probable. The tensile strength and fracture strain values found in this study are comparable to those found in studies published on FeMn alloys of similar composition [25,31,33,34]. The yield strength is even significantly higher than reported in literature. The high yield strength is attributed to the small grain size in agreement with the Hall–Petch relation [65,66]. Especially the existence of the very small, almost nano-scaled grains enhances the resistance to dislocation movement and, in turn, enhances the yield strength. High yield strength is a very desirable feature since thinner structures are sufficient in order to fulfill the mechanical requirements for an implant.

Besides improving the mechanical properties, it is the goal [25] to control the corrosion rate by alloying Fe with the less noble Mn and *U*_c_ to shift to more negative values. A number of studies proved that alloying with Mn leads to an increased degradation rate [25,26,28,31]. However, other studies [24,36,67,68] displayed contradictory results. Especially in vivo, the corrosion rate of FeMn seems to be slower compared to pure Fe. Even the results of the electrochemical corrosion measurements (Figure 5) show distinctly lower *j_c_* and CR values for the FeMn samples in comparison to the previously reported results for sputtered pure Fe. It is difficult to make a meaningful comparison with literature results since there is no standard procedure for the in vitro corrosion tests. Even if, in all works, it is attempted to mimic physiological conditions, there are still significant differences. Besides the used testing solution, the employed buffer system may have a significant influence. In this work, the pH was adjusted to 7.4 by a bicarbonate buffer system which includes a CO_2_ inlet. Mouzou et al. [56,69] showed that the type of degradation products of in vitro corrosion measurements strongly depends on the exact composition of the used electrolyte. Furthermore, they demonstrated that the CO_2_ content plays a key role. It was inferred that a CO_2_-rich environment favors the formation of Fe/MnCO_3_ layers. These layers were found to be Mn-rich. Thus, especially Mn-rich alloys under CO_2_-rich electrolyte tend to form carbonate passivation layers that hinder the degradation. This is assumed to be the explanation for the low degradation rates found in this study. However, to prove this assumption, further investigations regarding the degradation products are necessary. In comparison to studies that related the corrosion rate to the grain size [10,13], the differences in this study are up to two orders of magnitude smaller. It is inferred that the rather slight differences in the grain sizes are too small to significantly affect the passivation behavior and CR, respectively.

With respect to the application as biodegradable material, the low CR is not necessarily a drawback. Schinhammer et al. showed a decreasing biocompatibility of FeMn alloys with an increasing degradation rate. The effect was related to the increased release of Mn^2+^ ions into the testing medium [70]. Thus, in case of biodegradable FeMn alloys, a lower degradation rate could even be beneficial in terms of the biocompatibility, especially if the lower degradation rate is compensated by a high strength. Overall, the strength exceeds the values reported [31] for the SS 316L gold standard and in general values demanded in literature for biodegradable vascular implants (YS > 200 MPa, UTS > 300 MPa and A > 15–18%) [71]. The high yield strength of the presented material would allow thinner structures. The sample designing via UV-lithography offers great freedom in the device layout, which would allow the fabrication of filigree structured devices with a high relative surface. Therefore, less material would be required and the retention time of the implant could be reduced without increasing the amount of released Mn ions.

## 5. Conclusions

This study demonstrated that magnetron sputtering in combination with UV-lithography allows the fabrication of in situ structured FeMn foils. Hence, no further forming processes are necessary that might affect the microstructure or the phase composition and thus the material properties. The microstructure and phase composition of the foils are adjusted by heat treatment after the deposition in order to obtain a fine-grained homogeneous microstructure, resulting in a high strength and ductile material. The mechanical properties completely fulfill the requirements for biodegradable implants. The corrosion rate is lower compared to pure Fe. Furthermore, it is shown that the material exhibits an antiferromagnetic character, which is beneficial with respect to MRI compatibility. The saturation polarization is even lower compared to SS 316L used as FDA-approved material for medical implants.

This study opens the field for the further development of sputtered, biodegradable FeMn-based alloys. The method of sputtering allows a large number of further possibilities to enhance the degradation and mechanical properties. Considering the results found in this and the previous work on pure Fe, due to the microstructure of the sputtered material, superior mechanical properties can be expected.

## Figures and Tables

**Table 1:** Mean grain size $\bar{d}$, maximum grain size *d*_max_, and minimum grain size *d*_min_ of as-deposited and annealed FeMn samples.

Sample	$\bar{d}$ (nm)	*d*_max_ (nm)	*d*_min_ (nm)
FeMn as-deposited	451	2517	30
FeMn 800 °C, 2 h	965	3193	68
FeMn 950 °C, 2 h	1305	5078	143

**Table 2:** Magnetic Properties.

Sample	*J*_S_ (T)	*J*_R_ (T)	µ_0_H_C_ (mT)
Fe as-deposited	2.146	0.823	1.584
Fe 950 °C, 2 h	2.032	0.715	1.147
FeMn as-deposited	0.031	6.21 × 10^−3^	26.576
FeMn 800 °C, 2 h	1.09 × 10^−3^	0.13 × 10^−3^	14.752
FeMn 950 °C, 2 h	0.64 × 10^−3^	0.04 × 10^−3^	8.586
SS 316L	2.65 × 10^−3^	0.51 × 10^−3^	30.212
